# Impact of intrauterine fetal resuscitation with oxygen on oxidative stress in the developing rat brain

**DOI:** 10.1038/s41598-021-89299-w

**Published:** 2021-05-07

**Authors:** Jia Jiang, Tusar Giri, Nandini Raghuraman, Alison G. Cahill, Arvind Palanisamy

**Affiliations:** 1 Department of Anesthesiology, Washington University School of Medicine, St. Louis, MO 63110 USA; 2 Department of Obstetrics and Gynecology, Washington University School of Medicine, St. Louis, MO 63110 USA; 3 Department of Women’s Health, Dell Medical School, The University of Texas at Austin, Austin, TX USA; 4 Present Address: Department of Anesthesiology, Beijing Chaoyang Hospital, Capital Medical University, Beijing, China

**Keywords:** Neurological models, Cellular neuroscience, Neonatal brain damage

## Abstract

Use of maternal oxygen for intrauterine resuscitation is contentious because of the lack of evidence for its efficacy and the possibility of fetal harm through oxidative stress. Because the developing brain is rich in lipids and low in antioxidants, it remains vulnerable to oxidative stress. Here, we tested this hypothesis in a term pregnant rat model with oxytocin-induced fetal distress followed by treatment with either room air or 100% oxygen for 6 h. Fetal brains from both sexes were subjected to assays for biomarkers of oxidative stress (4-hydroxynonenal, protein carbonyl, or 8-hydroxy-2ʹ-deoxyguanosine), expression of genes mediating oxidative stress, and mitochondrial oxidative phosphorylation. Contrary to our hypothesis, maternal hyperoxia was not associated with increased biomarkers of oxidative stress in the fetal brain. However, there was significant upregulation of the expression of select genes mediating oxidative stress, of which some were male-specific. These observations, however, were not accompanied by changes in the expression of proteins from the mitochondrial electron transport chain. In summary, maternal hyperoxia in the setting of acute uteroplacental ischemia-hypoxia does not appear to cause oxidative damage to the developing brain.

## Introduction

Maternal oxygen administration is one of the most widely practiced interventions for intrauterine resuscitation of a distressed fetus^1–5^. However, whether such an intervention improves fetal or neonatal outcomes is questionable. Recent meta-analyses suggest that maternal oxygen does not improve either fetal oxygenation or acid–base status^3,5^. In addition, recent evidence from a randomized controlled trial suggested that room air resuscitation was non-inferior to oxygen therapy in the management of labor with Category II fetal heart rate tracing^6^. Taken together with the added concern that maternal oxygen therapy and the relative hyperoxic environment could increase plasma biomarkers of oxidative stress during delivery^4,7,8^, it is plausible that oxygen might be harmful rather than helpful. An unanswered question in this regard is whether the oxidative stress affects the developing fetal brain. This is important because the fetal brain is rich in lipids and low in antioxidants which could make it vulnerable to the effects of oxidative stress^9,10^. A meaningful inquiry in human subjects, however, is not possible because of ethical limitations. To address this knowledge gap, we utilized our pragmatic term pregnant rat model designed to induce fetal distress by stimulating aberrant uterine contractility with oxytocin (OXT) ^11^. Using this model, we investigated the effect of fetal resuscitation with either room air or 100% oxygen with the hypothesis that resuscitation with oxygen, compared to room air, would increase oxidative stress in the fetal brain. Furthermore, considering the sex-differences in the response to oxidative stress^12–16^, we speculated that the male brain would be especially vulnerable.

## Results

**Figure 1:**
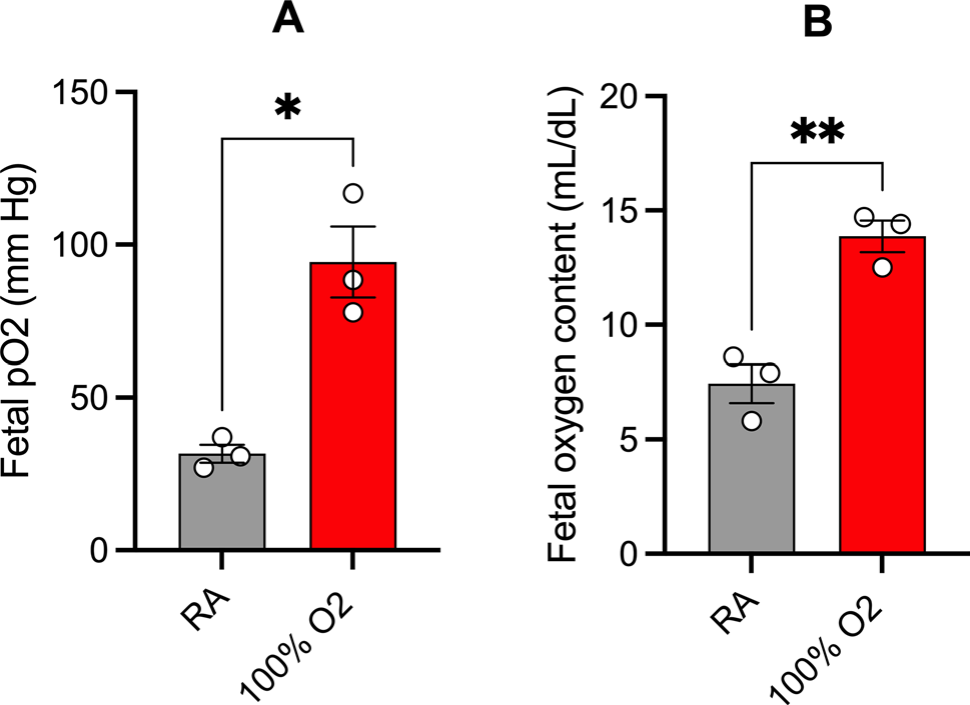
Maternal hyperoxia is associated with increased fetal oxygenation. Scatter plots showing the extent of increase in fetal PaO_2_ (**A**) and oxygen content (**B**) after maternal hyperoxia with 100% oxygen for 6 h. Data are presented as mean ± S.E.M and analyzed with Welch’s t-test; *p ≤ 0.05 and **p ≤ 0.01 (n = 3 per treatment condition).

Dams from both room air and oxygen groups tolerated the experiment and all pups were noted to be alive at the time of cesarean delivery. The number of dams per treatment condition and their litter data are shown in Table 1. We first assessed whether maternal hyperoxia was associated with increased fetal oxygenation. Maternal exposure to 100% oxygen was associated with a significant increase in the PaO_2_ and oxygen content of fetal left ventricular blood (Table 2 and Fig. 1). However, there were no significant differences in the pH, paCO_2_, HCO_3_, base deficit, or lactate levels between the two groups.

**Table 1:** Details of animal use.

	Control (RA)	Oxygen (100% O_2_)	P value
E 20 dams	8	8	
Litter size (mean ± S.D)	10.6 ± 2.4	11.4 ± 1.8	0.48
**Sex of the pups (mean ± S.D.)**			
M	4.5 ± 2.3	5.5 ± 1.8	0.35
F	6.1 ± 1.8	5.9 ± 1.6	0.77
Survival	100%	100%	

One male and female pup/unique dam/treatment condition was used for experiments unless stated otherwise, with the dam as the experimental unit. M: male offspring; F: female offspring.

**Table 2:** Blood gas analysis of the pups after in utero exposure to maternal hyperoxia.

	RA (n = 3)	100% O_2_ (n = 3)	p-value
pH	7.1 ± 0.04	7.2 ± 0.07	0.28
pO_2_ (mmHg)	33 ± 2.6	86 ± 12	0.02*
pCO_2_ (mmHg)	71 ± 7.3	55 ± 6.8	0.14
HCO_3_ (mmol/L)	22 ± 0.18	21 ± 1.7	0.71
Lactate (mmol/L)	12 ± 0.7	14 ± 1.1	0.21
Base excess (mmol/L)	− 8.2 ± 0.72	− 9.7 ± 1.4	0.41
O_2_ content (mL/dL)	6.3 ± 1.3	13 ± 0.73	0.006**

Left ventricular blood from three pups/unique dam/treatment condition was pooled prior to blood gas analysis, with the dam considered as the experimental unit.

*p ≤ 0.05; **p ≤ 0.01

**Figure 2:**
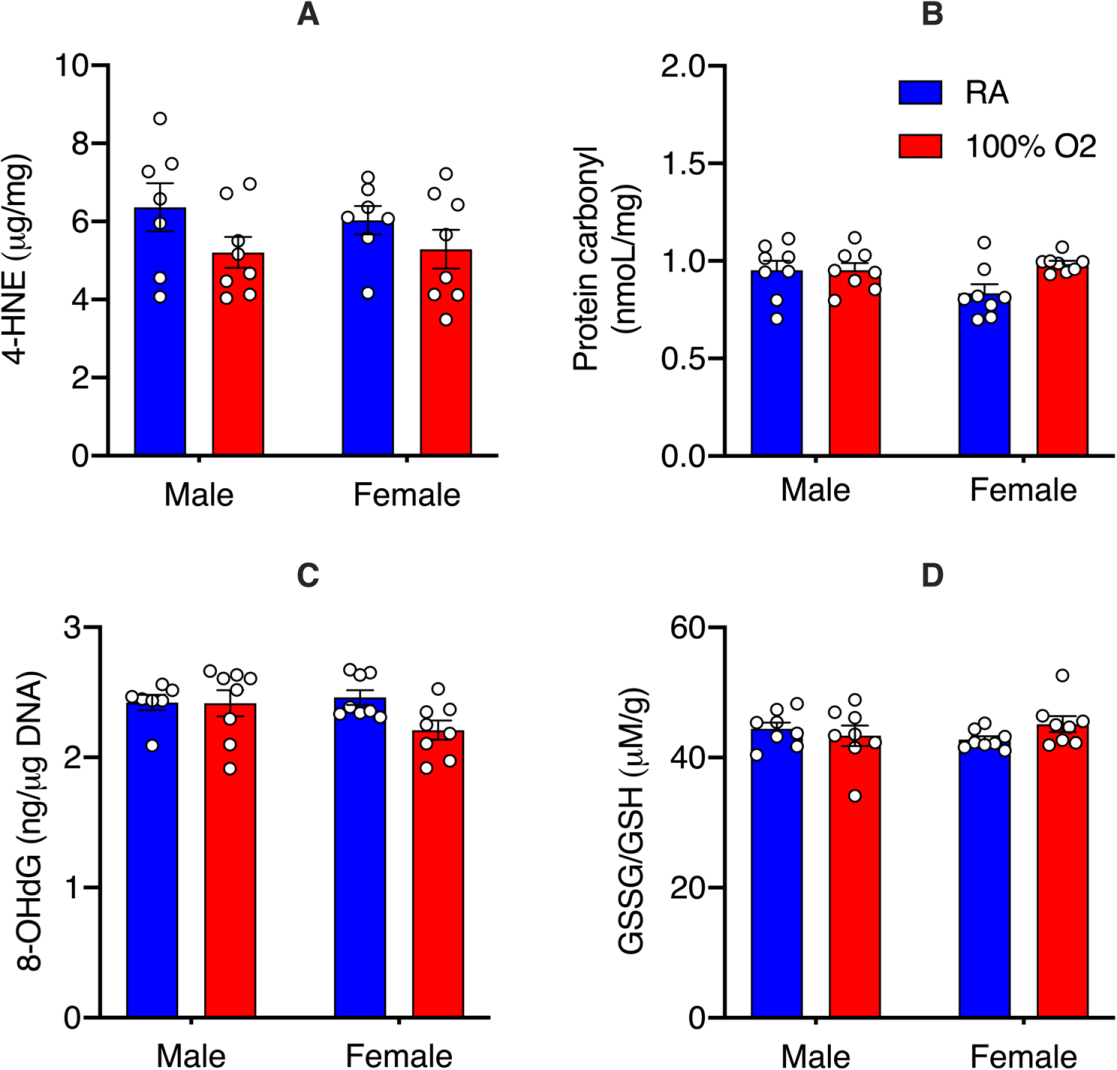
Oxidative stress biomarkers in the fetal brain after maternal hyperoxia. Scatter plots showing the extent of oxidative damage to lipids, proteins, and DNA as quantified by 4-hydroxynonenal (**A**), protein carbonyl (**B**), and 8-OHdG (**C**), respectively. Maternal hyperoxia was not associated with an increase in any of the biomarkers in either sex. The GSSG/GSH ratio (**D**), indicative of the collective glutathione antioxidant response, was no different between the groups. Data are presented as mean ± S.E.M and analyzed with 2-way ANOVA with Sidak’s multiple comparisons test to assess for sex differences in the treated group (n = 8 male and female pups from 8 unique dams/treatment condition).

We then assessed whether fetal resuscitation with maternal oxygen induces oxidative damage in the developing brain using validated biomarkers of oxidative stress (Fig. 2). We did not observe either treatment- or sex-specific differences in the concentration of 4-HNE, protein carbonyl, and 8-OHdG in the fetal cortex (Fig. 2A–C). Similarly, the magnitude of the antioxidant response, assessed with GSSG/GSH ratio was also no different between the treatment conditions of either fetal sex (Fig. 2D).

**Figure 3:**
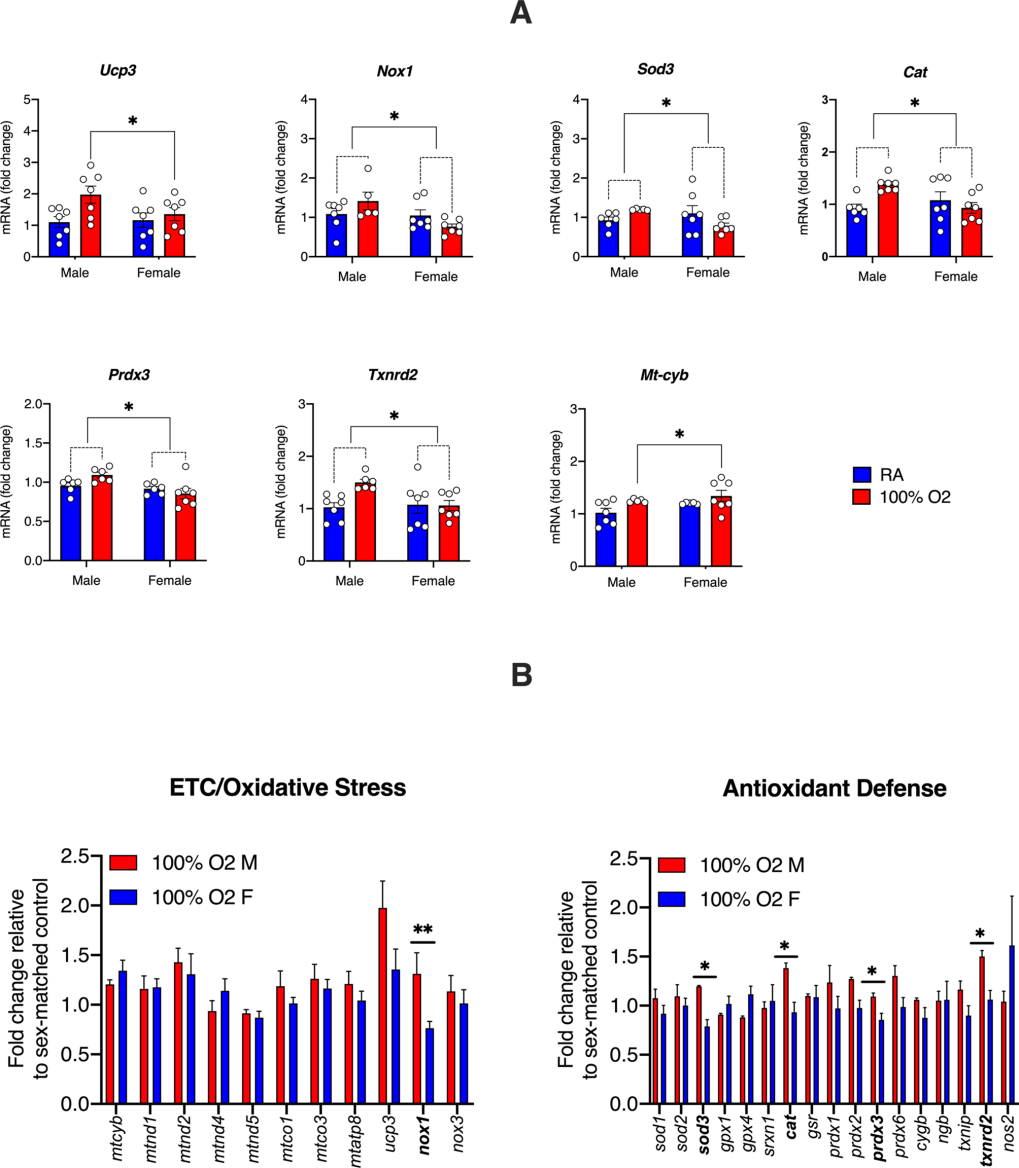
Differentially expressed oxidative stress genes in the fetal brain after maternal hyperoxia. (**A**) Scatter plots showing differential expression of genes involved in the oxidative stress response and prevention after maternal hyperoxia. There was a significant effect of treatment for *Ucp3* ([F (1, 24) = 5.7]) and *Mt-cyb* ([F (1, 20) = 5.06]), and a significant treatment *vs*. sex interaction for *Nox1* ([F (1, 22) = 4.32]), *Sod3* ([F (1, 21) = 5.5]), *Cat* ([F (1, 23) = 7.79]), *Prdx3* ([F (1, 21) = 4.46]), and *Txnrd2* ([F (1, 23) = 4.76]). (**B**) Bar graphs highlighting the sex-dependent differences in gene expression involving oxidative stress (*left*) and antioxidant defense (*right*) after maternal hyperoxia with 100% oxygen (ETC: electron transport chain). Data are presented as mean ± S.E.M and analyzed with 2-way ANOVA with Sidak’s multiple comparisons test to assess for sex differences in the treated group; *p ≤ 0.05 and **p ≤ 0.01 (n = 7 male and female pups from 7 unique dams/treatment condition).

We next examined the impact of fetal resuscitation with oxygen on the differential expression of genes associated with oxidative stress and mitochondrial oxidative phosphorylation in the developing brain of both sexes (Fig. 3). Unlike the oxidative stress biomarkers, we observed a significant upregulation in the expression of genes mediating oxidative stress (*Ucp3*, *Nox1*), antioxidant response (*Sod3*, *Cat*, *Prdx3*, *Txnrd2*) and oxidative phosphorylation (*Mt-cyb*) in the developing brain after oxygen exposure (Fig. 3A). Among these genes, *Nox1*, *Sod3*, *Cat*, *Prdx3*, and *Txnrd2* were differentially upregulated in the male *vs*. female brain after resuscitation with oxygen (Fig. 3B).

**Figure 4:**
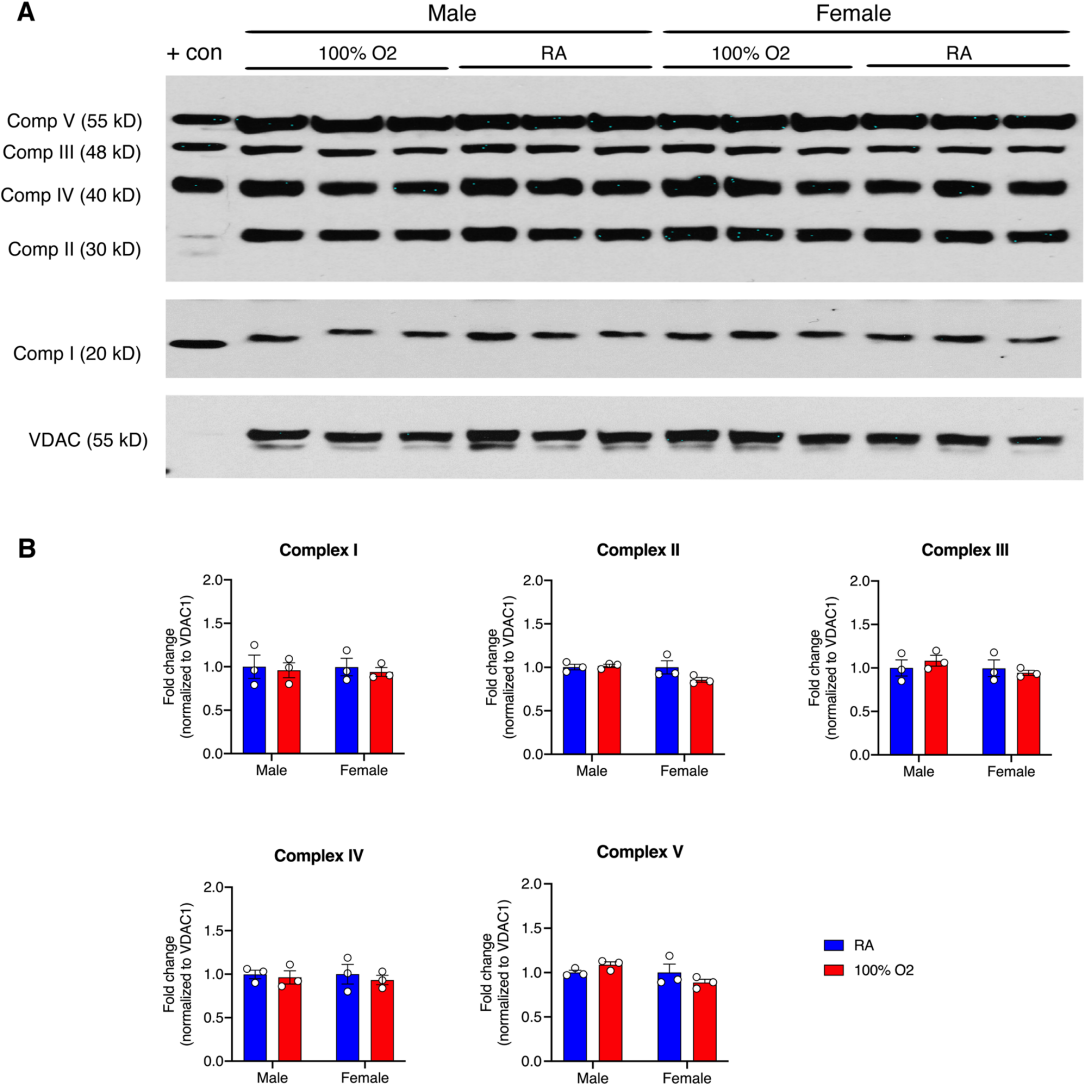
Changes in mitochondrial oxidative phosphorylation in the fetal cortex after maternal hyperoxia. (**A**) Representative immunoblots showing all 5 electron transport chain complexes in the cerebral cortical homogenates of both male and female offspring after maternal exposure to either room air or hyperoxia, with rat heart mitochondria as positive control. Exposure time had to be increased from 1 to 3 min to visualize Complex I. (**B**) Scatter plots highlighting the lack of significant changes in mitochondrial OXPHOS proteins in the fetal brain after exposure to 6 h of maternal hyperoxia with 100% oxygen. Data are presented as mean ± S.E.M and analyzed with 2-way ANOVA with Sidak’s multiple comparisons test to assess for sex differences in the treated group (n = 3 each for all experiments).

Considering our previous work showing changes in mitochondrial biogenesis after acute intrapartum hypoxemia^11^, we were interested in understanding the additive impact of maternal administration of oxygen on mitochondrial oxidative phosphorylation. There were neither treatment nor sex-related differences in the expression of proteins related to any of the mitochondrial electron transport chain complexes in the maternal hyperoxia group (Fig. 4).

## Discussion

Our investigations in a term pregnant rat model show that maternal hyperoxia in the setting of induced fetal distress did not appear to be associated with oxidative damage to the developing brain. Though we observed male sex-specific upregulation of select genes mediating oxidative stress and antioxidant defense, this was not accompanied by any changes in the mitochondrial proteins involved in the oxidative phosphorylation pathway. Taken together with our previous data showing a significant effect of acute uteroplacental ischemia on oxidative stress and behavioral outcomes even with room air treatment^11^, our results suggest that the magnitude of the initial hypoxemic insult is more likely to impact outcomes rather than the choice of oxygen or room air resuscitation.

Our targeted preclinical research provides important novel data on oxidative stress in the fetal brain during intrauterine resuscitation with maternal oxygen after placental ischemia-hypoxemia. Though maternal hyperoxia is known to increase biomarkers of oxidative stress in the umbilical cord blood^7,17^, it is unclear whether the putative oxidative stress affects the fetal brain, arguably the most important organ of interest. By adopting a pragmatic model of acute, reversible uteroplacental ischemia caused by oxytocin-induced aberrant uterine contractility, our studies were designed to reflect a relatively common and clinically relevant scenario during labor. The absence of oxidative damage in the fetal brain after maternal hyperoxia was contrary to our hypothesis. This unexpected result could partly be explained by dynamic changes in fetal cerebral blood flow and oxygenation during acute ischemia-hypoxia. For example, acute hypoxemia followed by reperfusion and a relatively hyperoxic environment is likely to result in enhanced oxidative stress. So, what could protect the fetal brain from maternal hyperoxia? Preserved ‘brain-sparing’ blood flow in a previously uncompromised fetus could reduce the fetal brain impact of acute uteroplacental ischemia and minimize oxidative stress by reducing the magnitude of initial hypoxemia^18^. Conversely, it is equally plausible that oxygen delivery to the brain is not significantly enhanced after maternal hyperoxia, thereby limiting the generation of oxygen free radicals. Support for this possibility comes from elegant BOLD MRI imaging studies in pregnant subjects showing that maternal hyperoxia does not increase the signal in the fetal brain despite an increase in signal in the extra-cranial fetal organs^19^. Furthermore, there is evidence that maternal hyperoxia might directly increase fetal cerebral vascular resistance and redistribute blood flow away from the brain^20,21^. However, unlike the compromised pups in utero in our study, these imaging studies were performed in normoxic fetuses at baseline, which might influence the magnitude and direction of the ‘brain-sparing’ effect. Finally, humoral responses typically associated with altered vasoreactivity after hyperoxia, such as decreased production of nitric oxide and inhibition of endothelial prostaglandin synthesis observed in other settings, could be at play^22^. Taken together, we are convinced that, unlike the well-characterized fetal consequences of maternal hypoxia, the fetal effects of maternal hyperoxia are nuanced and need more targeted mechanistic investigations. Nevertheless, we are reassured with the observation that maternal hyperoxia in the setting of fetal compromise does not increase oxidative stress biomarkers in the developing brain.

The substantial upregulation of genes related to both the oxidative stress and the antioxidant pathway after maternal hyperoxia is a novel finding. We could not ascertain whether the antioxidant response was commensurate with the degree of oxidative stress. However, considering the relatively benign impact of maternal hyperoxia on oxidative stress biomarkers, we are inclined to favor the possibility of a proportionate antioxidant response. More interesting to us was the sex difference in the oxidative stress response, with male fetuses showing a marked upregulation of *Nox1* gene, accompanied by a substantial increase in antioxidant enzymes suggestive for a compensatory response. This is perhaps not surprising considering the sex differences in oxidative stress response, mitochondrial biology, and gonadal steroid milieu in the developing brain^23–27^. For example, females appear to have increased intracellular glutathione^28^, increased level of the antioxidant paraoxonase 2^29^, and more robust mitochondrial biogenesis in males possibly predisposing them to oxidative stress^30^. A majority of these changes, however, are known to be mediated by estradiol in the post-pubertal brain, so it remains unclear if the hormonally neutral intrauterine environment results in such changes in the redox environment of the developing brain. The male-specific upregulation of *Nox1* (NADPH oxidase 1) warrants further studies because of its integral role as a dedicated mammalian enzyme system involved in the generation of superoxide anions^31^, a major form of reactive oxygen species. Upregulation of *Nox1* is widely observed during recovery from ischemic stroke^32–34^, suggesting that there could be mechanistic parallels between the impact of acute intrapartum ischemia-hypoxemia on the fetal brain and ischemic stroke. Future studies are required to determine whether these redox responses are persistent and enduring, and whether they lead to functionally variant outcomes in the offspring.

We had previously shown that mitochondrial oxidative phosphorylation was permanently upregulated in the cingulate cortex of adolescent male, but not female, rat offspring after uteroplacental ischemia induced by aberrant uterine contractility even without maternal hyperoxia^11^. Considering the changes in oxidative stress gene expression with maternal hyperoxia, we sought to determine if this was accompanied by changes in mitochondrial oxidative phosphorylation. Though we limited our investigations to the immediate post-hyperoxia period, we did not observe related changes in the expression of proteins from the mitochondrial electron transport chain. Whether these sex-dependent changes in oxidative stress gene expression are consequential, enduring, and cause altered neurobehavioral outcomes need to be determined.

The biggest strength of the study is that it provides the basic scientific foundation regarding the impact of maternal hyperoxia on oxidative stress in the fetal brain. By replicating an acute in utero clinical scenario, our study is distinct from other studies in the field that investigate the effect of hypoxia in newborn pups. The only comparable study is by Boksa et al. which reported a nuanced effect of oxytocin on redox biology in the fetal brain^35^, oxytocin increased the lactate levels but reduced the concentration of brain malondialdehyde, an oxidative stress marker. Though reduced oxidative stress may appear to be a counter-intuitive observation, oxytocin was administered in this study as a continuous, low-dose infusion which presumably had negligible effects on uteroplacental perfusion, and therefore fetal oxidative stress, compared to the acute placental ischemia-hypoxemia noted with our model. Furthermore, it is unclear whether such changes were caused by oxytocin per se or the 6–15 min of induced anoxia at birth that was part of the experimental paradigm. In addition, a direct effect of oxytocin could not be ruled out. In contrast, our previously established paradigm clearly demonstrated significant uteroplacental ischemia by inducing uterine hypercontractility with a dose of oxytocin that is not known to cross the placenta^11,36^, resulting in increased confidence in our results.

Our study needs to be interpreted in the context of a few limitations. First, the lack of assessment of blood gases in the dam, and therefore, confirmation of maternal hyperoxia, could be cited as a limitation. However, previous data from our lab had shown that maternal PaO_2_ was approximately 350 mmHg after 4 h of 100% oxygen in spontaneously breathing dams^37^, providing reassurance that maternal hyperoxia was achieved. The more relevant unanswered question was whether maternal hyperoxia was associated with fetal hyperoxia which we addressed comprehensively with blood gas studies showing increased partial pressure of oxygen and oxygen content in the fetal circulation. Second, our assessment time point of 6 h may have been either too early or too late resulting in a failure to capture the magnitude of oxidative stress. However, corroborative evidence suggests that biomarkers of oxidative damage such as 4-HNE and protein carbonyl can be detected as early as 1–3 h and can be elevated up to 12 h^38–41^, rendering that possibility unlikely. If 6 h of maternal hyperoxia does not cause oxidative damage, it is probably safe to assume that short-term administration of oxygen in the clinical setting is unlikely to have a major impact. Third, we did not perform histological analysis of the fetal brain to determine if there was neuronal cell death. Though we did not observe neuronal apoptosis after placental ischemia-hypoxemia in our previous study^11^, direct exposure to 100% oxygen can be neurotoxic to the developing brain especially during prolonged or repetitive exposure^42,43^, and therefore, needs to be ascertained in future experiments. Third, our study does not exclude the possibility of oxidative stress in fetal organs other than the brain. Similarly, a brain-region specific change in oxidative stress could not be investigated because of the difficulty in ensuring accurate anatomic brain dissection at this developmental age. Finally, the lack of functional neurobehavioral outcomes might be considered a limitation, but our study was primarily designed to be a molecular biological examination of the developing brain after in utero exposure to maternal hyperoxia.

In summary, we report that there is no major oxidative damage to the fetal brain after maternal oxygen therapy in the setting of intrapartum fetal compromise. Although we noted significant differences in the expression of genes in the oxidative stress-mitochondrial oxidative phosphorylation pathway, of which some were sex-dependent, there were no changes in the expression of emblematic proteins from the mitochondrial oxidative phosphorylation pathway. Collectively, our data provide foundational knowledge to better understand the impact of the choice of resuscitative agent for fetal distress and raises the biological possibility that sex-specific differences in neonatal neurological outcomes could potentially be amplified by the choice of maternal resuscitative agent. Therefore, follow up studies are required to determine if these gene expression changes are persistent and whether they lead to worsening of functional neurobehavioral outcomes.

## Materials and methods

All experimental procedures were approved by the Institutional Animal Care and Use Committee at Washington University in St. Louis (#20170010) and comply with the Animal Research: Reporting of In Vivo Experiments (ARRIVE) guidelines. All methods proposed here were performed in accordance with relevant institutional guidelines and regulations.

### Animals and treatment

Timed pregnant Sprague–Dawley (SD) rats (SAS 400, Charles River Laboratories, Wilmington, MA) were acquired on embryonic day (E) 13 and housed under standard housing conditions until experimentation. On E20, randomized dams were administered 100 mcg/kg oxytocin (OXT) (1 mg/mL in sterile normal saline, Selleck Chemicals, Houston, TX) through a 25G tail vein catheter under brief isoflurane anesthesia (with compressed 21% oxygen as the carrier gas) to induce a 10 min tetanic uterine contraction and acute placental ischemia-hypoxemia as described by us previously^11^. In this paradigm, fetal distress was confirmed with elevated fetal brain lactate. We chose this dose of OXT because of minimal transplacental transfer^36^, thereby excluding the possibility of a direct effect of OXT on the developing brain. Following treatment, dams were immediately randomly assigned to two groups during the ischemia–reperfusion period: room air (RA) and 100% oxygen (O). In group RA, treated dams (n = 8) were exposed to 21% oxygen administered at a flow rate of 3L/min into a standard rat container (Kent Scientific Corporation, Torrington, CT) for 6 h. Similarly, in Group O, dams (n = 8) were exposed to 100% oxygen at the same flow rate for 6 h. All containers (3 L total volume) were pre-filled with the respective gases at a rate of 5L/min for at least 3 min to reach the satisfied oxygen concentration. A small outlet in the container allowed for monitoring of gases and prevented buildup of carbon dioxide. All dams tolerated the treatment with OXT and the subsequent exposure to different gases. 6 h after either RA or O exposure, fetuses were removed via cesarean delivery under brief isoflurane anesthesia (in 21% oxygen for group RA and 100% oxygen for group O, respectively, to maintain treatment group-specific oxygen concentration). Pups were quickly sexed based on differences in anogenital distance between males and females, and both male and female brains were extracted and snap-frozen in liquid nitrogen for storage at − 80 °C. One pup of either sex was used per treatment condition.

### Collection of fetal left ventricular blood for blood gas analysis

To assess whether maternal hyperoxia was associated with an increase in fetal oxygenation, we performed a separate set of experiments. Briefly, 6 E20 timed-pregnant Sprague Dawley dams were administered 100 mcg/kg OXT through a 25G tail vein catheter followed immediately by random assignment to either room air or 100% oxygen treatment (n = 3 each) for 6 h as described above. Subsequently, at least 3 fetuses were collected per dam via cesarean delivery and immediately dissected to access the thoracic cavity. Because left ventricular puncture was technically challenging due to the rapid heart rate and the extremely low residence time of blood, we transected the left ventricle, allowed the blood to pool in the thoracic cavity, and aspirated it immediately into a heparin-coated 1 mL syringe. We were able to collect approximately 100 µL per fetus, and the entire 300 µL from all three fetuses per dam was thoroughly admixed in the same syringe before blood gas analysis with the Stat Profile Prime^®^ Analyzer (Nova Biomedical, Waltham, MA).

### Assays for biomarkers of oxidative stress

To assess whether exposure to maternal hyperoxia causes oxidative damage to the fetal brain, we first assayed for biomarkers of oxidative stress. Specifically, we investigated oxidative damage to lipids (4-hydroxynonenal, a lipid peroxidative product), proteins (protein carbonyl), and DNA (8-hydroxy-2′-deoxyguanosine [8-OHdG]) in the brains of both sexes. In addition, we assayed for the glutathione (GSSG/GSH) ratio as a marker of the antioxidant response. Approximately 25–50 mg of brain tissue from the left cerebral cortex was used for all experiments. Protein concentration was determined using Pierce™ BCA Protein Assay Kit (ThermoFisher Scientific). The extent of oxidative damage to lipids and proteins was assessed with OxiSelect™ HNE Adduct Competitive ELISA and OxiSelect™ Protein Carbonyl ELISA kits (Cell Biolabs), respectively, according to manufacturer’s instructions but with slight modifications (addition of PEI to a final concentration of 0.5% for the protein carbonyl assay). 8-OHdG was quantified in specially prepared cortical tissue lysates. Briefly, approximately 25 mg of cortical tissue was homogenized to powder with liquid nitrogen using a pestle. DNA was extracted using the DNeasy Blood and Tissue Kit (Qiagen, USA) and the concentration was measured using a NanoDrop 2000 spectrophotometer (Thermo Scientific™, USA). Oxidative DNA damage marker 8-oxoguanine (8-OHdG) was quantified using the OxiSelect™ Oxidative DNA Damage ELISA kit (Cell Biolabs) according to manufacturer’s instructions. Absorbance for all assays was measured at 450 nm. For the total glutathione (GSSG/GSH) assay, lysates were prepared with approximately 50 mg of cortical tissue treated with 5% metaphosphoric acid followed by quantification with OxiSelect™ Total Glutathione (GSSG/GSH) Assay kit (Cell Biolabs) according to manufacturer’s instructions. Absorbance was immediately read at 405 nm at 1 min intervals for 10 min to determine the slopes for final calculations.

### Taqman RT-qPCR for differential expression of oxidative stress genes

Total RNA was isolated from the right fetal cerebral cortex (approx. 30 mg) using RNeasy Mini Kit (Qiagen, Germantown, MD). Genomic DNA was eliminated using QIAshredder (Qiagen, Germantown, MD) and an on-column treatment with RNase-Free DNase Set (Qiagen, Germantown, MD). 2.5 µg of purified RNA (260/280 ratio ≥ 2.0) was reverse transcribed to cDNA using SuperScript™ IV VILO™ Master Mix with ezDNase™ Enzyme (ThermoFisher Scientific, Waltham, MA) in a final volume of 20 μl at 37 °C for 30 min, 50 °C for 10 min, 85 °C for 5 min, and 4 °C for 30 min. Subsequently, we performed planned comparisons of treatment-related differences in the expression of select target genes related to oxidative stress (*Nox1, Nox3, Nos2, Ucp3),* antioxidant defense (*Cat, Gpx1, Gpx4, Gsr, Prdx1, Prdx2, Prdx3, Prdx6, Sod1, Sod2, Sod3, Srxn1, Cygb, Ngb, Txnip, Txnrd2*), and the mitochondrial electron transport chain (ETC) (*Mt-cyb, Mt-nd1, Mt-nd2, Mt-nd4, Mt-nd5, Mt-atp8, Mt-co1, Mt-co3*). We included mitochondrial ETC genes because of the integral role of mitochondria in the generation of reactive oxygen species during oxidative stress. A customized TaqMan Array 96-well FAST plate (ThermoFisher Scientific, Waltham, MA) containing these 28 prevalidated probes and 4 endogenous genes (*18S rRNA, Actb, Gapdh, Pgk1*) was used for qPCR. Each 10 μl reaction contained 5 μl of TaqMan Master Mix (ThermoFisher Scientific, Waltham, MA), 3μL of ultrapure water, and 2 μl of cDNA. qPCR was performed in an Applied Biosystems 7500 Real-Time PCR System (ThermoFisher Scientific, Waltham, MA) with the following cycling conditions: preincubation at 50 °C for 2 min and then at 95 °C for 10 min, followed by 40 amplification cycles (95 °C, 15 s and 60 °C, 1 min) and cooling (40 °C, 10 s). All reactions were performed in triplicate. Of the 4 endogenous reference genes, only *Actb* and *Gapdh* were stably expressed across experimental conditions (geNorm algorithm; qbase + version 3.2). Relative mRNA expression, normalized to the geometric mean of *Actb* and *Gapdh*, was calculated using the 2^−ΔΔCT^ method with sex-matched control samples as reference.

### Western blot for mitochondrial oxidative phosphorylation

Next, we performed immunoblots of the developing cortex to quantify the effect of maternal oxygen resuscitation on the mitochondrial oxidative phosphorylation system (OXPHOS) in the developing brain. Briefly, we isolated mitochondria from approximately 100 mg of the fetal cortex using the Mitochondria Isolation Kit (ThermoFisher Scientific) followed by lysis and homogenization with RIPA buffer containing protease inhibitor and phosphatase inhibitor in 1X PBS. Protein concentrations were determined using a BCA protein assay kit (ThermoFisher Scientific, USA). 15 µg of each sample was treated with reduced LDS buffer and heated at 50 °C for 5 min, then loaded onto a 10% Bolt gel (ThermoFisher Scientific). Rat heart mitochondria was used as positive control. After separation in MES SDS running buffer (ThermoFisher Scientific), proteins were transferred to nitrocellulose membrane and subsequently blocked for 2 h at room temperature in 5% non-fat dry milk and 1X Tween 20 in Tris-buffered saline (TBS-T). Membrane was incubated with primary mouse anti-OXPHOS antibody (OriGene; 1:3000 diluted in 5% BSA and 1X TBS-T) overnight at 4 °C, followed by HRP-conjugated anti-mouse secondary antibody (1:5000 diluted in 5% non-fat dry milk and 1X TBS-T) for 1 h at room temperature. A chemiluminescent detection reagent (ECL Prime, GE Healthcare) was used to visualize the proteins. After stripping for 45 min followed by blocking with 5% non-fat dry milk and 1X TBS-T for 30 min, the membrane was reprobed with rabbit anti-VDAC1 antibody (Cell Signaling, US. 1:5000 diluted in 5% non-fat dry milk and 1X TBS-T) for 1 h at room temperature, followed by HRP-conjugated anti-rabbit secondary antibody (1:5000 diluted in 5% non-fat dry milk and 1X TBS-T) for 1 h at room temperature. Images were subsequently processed with Image Studio ver 5.2 (LI-COR) for densitometric quantification. Full-length western blot images are presented as Supplementary Information.

### Statistical analysis

Data outliers were detected and eliminated using ROUT (robust regression and outlier analysis) with Q set to 10%. Normality of residuals was checked with D’Agostino-Pearson omnibus test followed by Welch’s t-test for blood gas data and the 2-way ANOVA and Sidak’s multiple comparisons test for all other data where sex of the offspring was a variable. RT-qPCR data with non-normal residuals (*Nox1, Nos2, Prdx3, Sod3, Srxn1, Mt-atp8, Mt-co3*) were Box-Cox transformed prior to statistical testing. Data are presented as mean ± SEM and analyzed with Prism 8 for Mac OS X (Graphpad Software, Inc, La Jolla, CA). A two-tailed P value ≤ 0.05 was accorded statistical significance.

### Conference presentation

This abstract was presented at the 40th Annual Pregnancy Meeting, Society for Maternal Fetal Medicine, Grapevine, TX, 76501, February 3–8, 2020.

## Supplementary Information

Supplementary Information.
