# Supplementary material for: Impact of intrauterine fetal resuscitation with oxygen on oxidative stress in the developing rat brain
Source: Sci Rep. 2021 May 7;11:9798. doi: 10.1038/s41598-021-89299-w (PMC8105387; doi:10.1038/s41598-021-89299-w)
Supplement: Supplementary file 1 — Supplementary Information. [file 41598_2021_89299_MOESM1_ESM.docx]

**Impact of intrauterine fetal resuscitation with oxygen on oxidative stress in the developing rat brain**

Jia Jiang^1, #a^, Tusar Giri^1^, Nandini Raghuraman^2^, Alison G. Cahill^3^, Arvind Palanisamy^1,2*^

^1^ Department of Anesthesiology

Washington University School of Medicine

St. Louis, MO, 63110

^2^ Department of Obstetrics and Gynecology

Washington University School of Medicine

St. Louis, MO, 63110

^3^ Department of Women’s Health

The University of Texas at Austin

Dell Medical School, Texas

**Current address**

^#a^ Department of Anesthesiology, Beijing Chaoyang Hospital, Capital Medical University, Beijing, China

**Conflict of interest:** The authors report no conflict of interest.

**Funding:** Departmental startup funds to AP

**Paper presentation information:** This abstract was presented at the 40^th^ Annual Pregnancy Meeting, Society for Maternal Fetal Medicine, Grapevine, TX, 76501, February 3-8, 2020.

**^*^Corresponding author**

Arvind Palanisamy, MD, FRCA

Department of Anesthesiology

Washington University School of Medicine, St. Louis, MO, 63110

Tel: 314-362-2628

Email: [arvind.palanisamy@wustl.edu](mailto:arvind.palanisamy@wustl.edu)

**Supplementary Information**

**Full-length blot for Fig 4**

Low exposure – 1 min

**
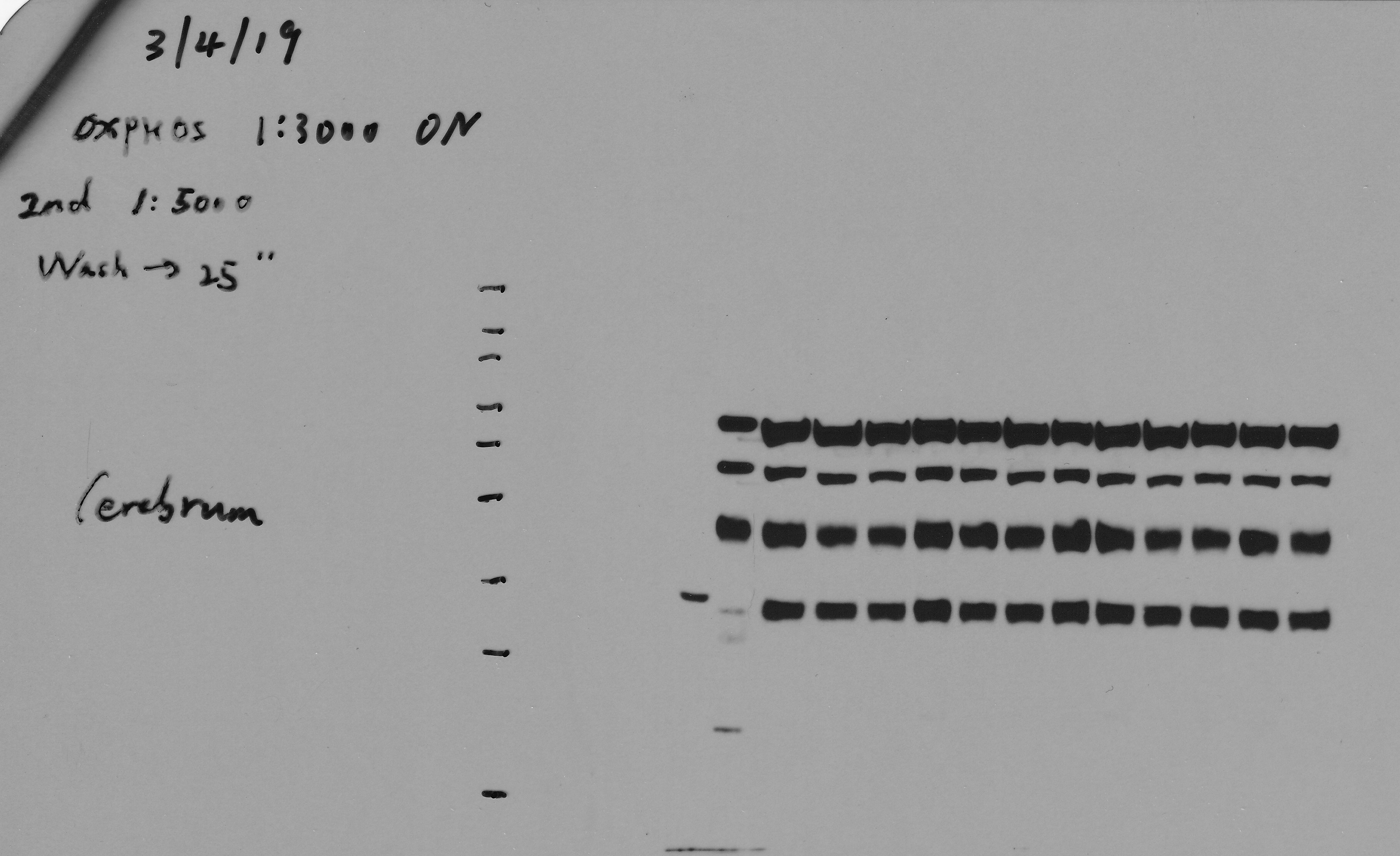
**

High exposure – 3 min (for Complex I)


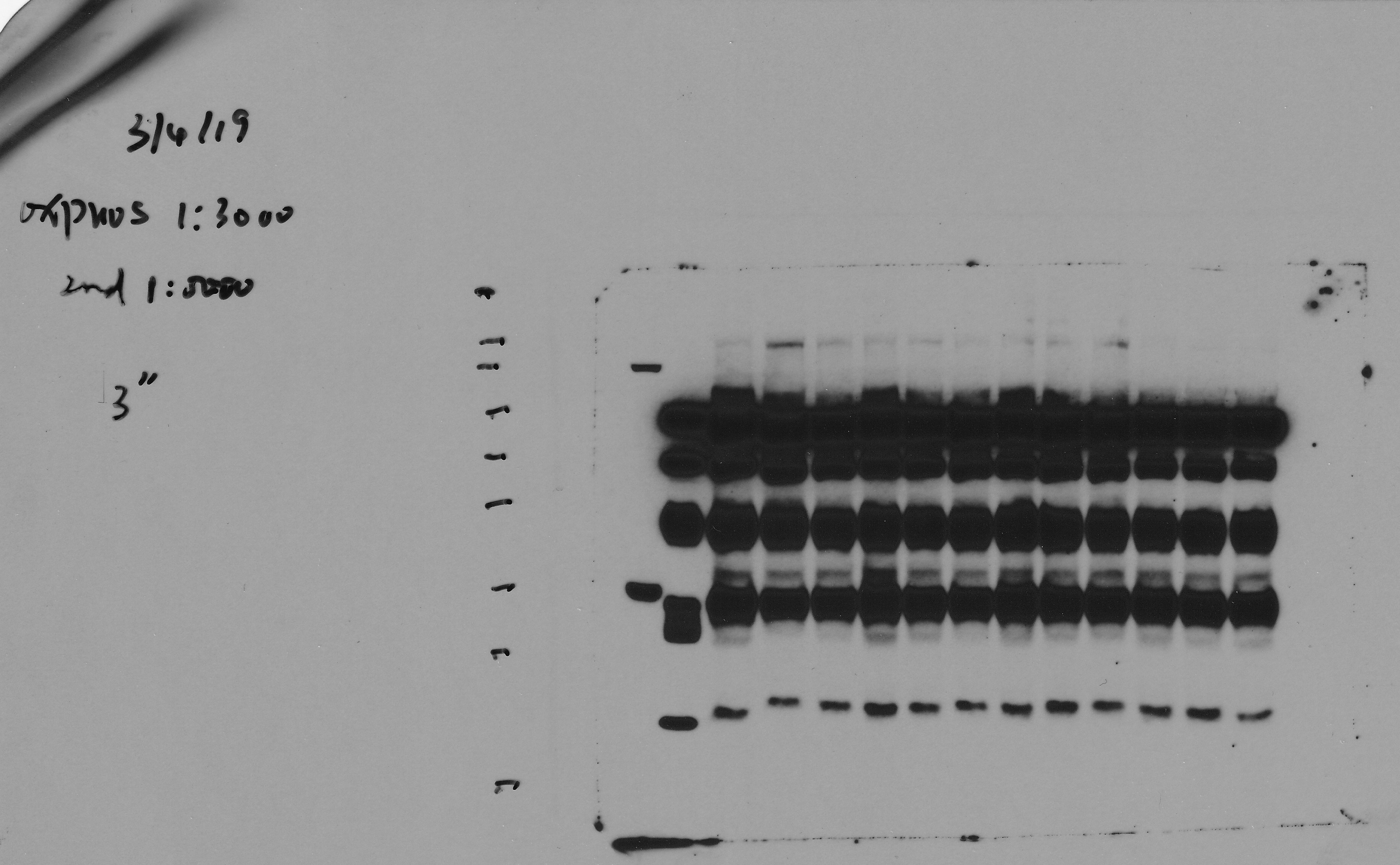


Comp V

Comp III

Comp IV

Comp II

High exposure film- 3 min

Very low (5 sec) exposure for VDAC


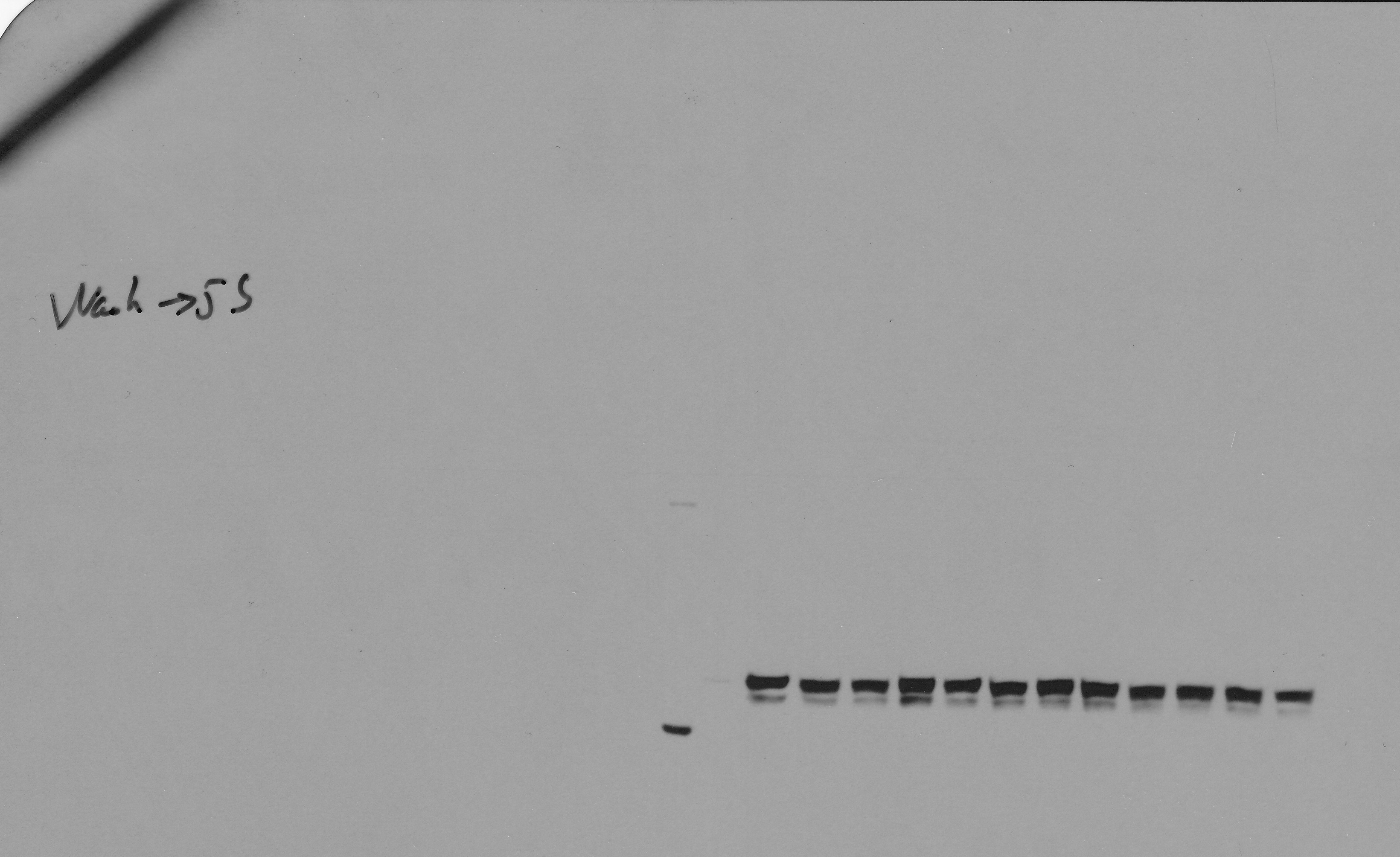


VDAC (55 kD)

Low exposure film- 5 sec
